# Advances in the Identification of Circular RNAs and Research Into circRNAs in Human Diseases

**DOI:** 10.3389/fgene.2021.665233

**Published:** 2021-03-19

**Authors:** Shihu Jiao, Song Wu, Shan Huang, Mingyang Liu, Bo Gao

**Affiliations:** ^1^Hainan Key Laboratory for Computational Science and Application, Hainan Normal University, Haikou, China; ^2^Yangtze Delta Region Institute (Quzhou), University of Electronic Science and Technology of China, Quzhou, China; ^3^Director of Preventive Treatment of Disease Centre, Qinhuangdao Hospital of Traditional Chinese Medicine, Qinhuangdao, China; ^4^Department of Neurology, The Second Affiliated Hospital, Harbin Medical University, Harbin, China; ^5^Department of Internal Medicine-Oncology, Heilongjiang Province Land Reclamation Headquarters General Hospital, Harbin, China; ^6^Department of Radiology, The Second Affiliated Hospital, Harbin Medical University, Harbin, China

**Keywords:** circRNAs, database, machine learning, circRNAs identification, diseases

## Abstract

Circular RNAs (circRNAs) are a class of endogenous non-coding RNAs (ncRNAs) with a closed-loop structure that are mainly produced by variable processing of precursor mRNAs (pre-mRNAs). They are widely present in all eukaryotes and are very stable. Currently, circRNA studies have become a hotspot in RNA research. It has been reported that circRNAs constitute a significant proportion of transcript expression, and some are significantly more abundantly expressed than other transcripts. CircRNAs have regulatory roles in gene expression and critical biological functions in the development of organisms, such as acting as microRNA sponges or as endogenous RNAs and biomarkers. As such, they may have useful functions in the diagnosis and treatment of diseases. CircRNAs have been found to play an important role in the development of several diseases, including atherosclerosis, neurological disorders, diabetes, and cancer. In this paper, we review the status of circRNA research, describe circRNA-related databases and the identification of circRNAs, discuss the role of circRNAs in human diseases such as colon cancer, atherosclerosis, and gastric cancer, and identify remaining research questions related to circRNAs.

## Introduction

**FIGURE 1:**
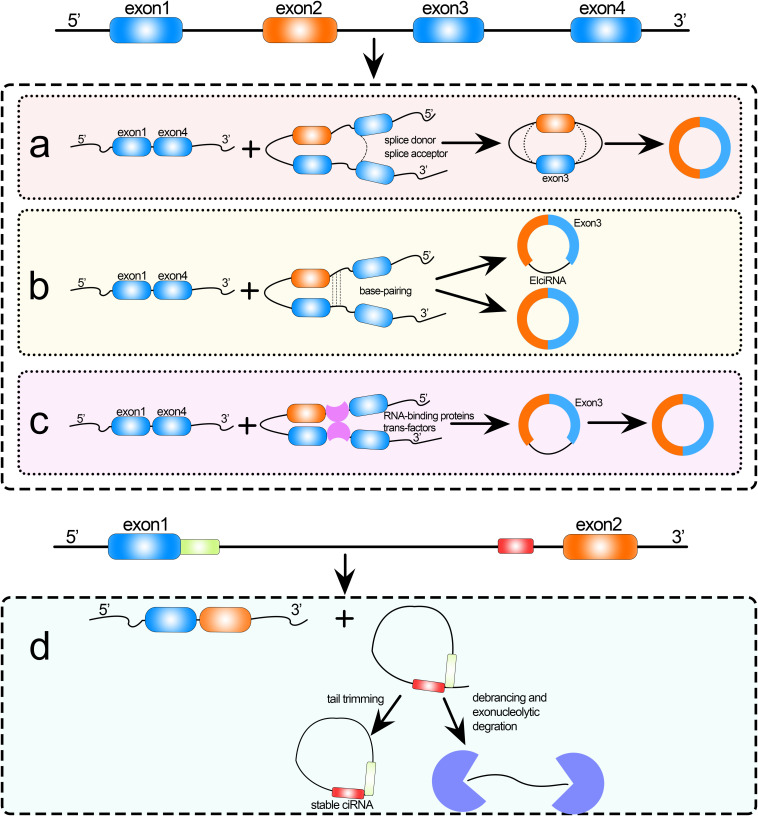
Formation of circRNAs by **(a)** exon skipping, **(b)** direct back-splicing, **(c)** formation by RNA-binding proteins (RBPs), and **(d)** circular intron RNA cyclization.

Circular RNAs (circRNAs) are endogenous non-coding RNAs (ncRNAs) that have gained increasing attention in recent years. circRNAs are formed by exon or intron cyclization that ligates the 5′ terminal cap and 3′ terminal poly(A) tail to form a circular structure. They are mainly located in the cytoplasm or stored in exosomes, are unaffected by RNA exonucleases, are more stably expressed and less susceptible to degradation, and have been shown to exist in a wide variety of eukaryotic organisms (Li Y. et al., 2015; Pradeep et al., 2020). The widespread existence of circRNAs suggests that they have certain biological functions as lncRNAs and microRNAs (miRNAs) play (Jiang et al., 2009, 2014, 2015; Wang et al., 2014; Cheng L. et al., 2019; Liang et al., 2019; Wei and Liu, 2020; Yang et al., 2020). In recent years, studies have shown a diversity of formation mechanisms and biological functions of circRNAs. circRNAs are formed by various mechanisms; for example, spliceosomes (intracellular protein–RNA complexes) catalyze splicing as follows (Salgia et al., 2003): first, the spliceosome recognizes introns, which are flanked by the splice donor (or 5′ splice site) and the splice acceptor (or 3′ splice site) with specific sequences at the 5′ and 3′ ends; then, the 2′ hydroxyl group of the downstream sequence attacks the splice donor, resulting in a circular intron lariat structure; finally, the 3′ hydroxyl group of the upstream exon splice donor attacks the splice acceptor, the upstream and downstream exons are sequentially spliced to form a linear structure, and the intron lariat structure is usually degraded rapidly by debranching enzyme. Variable splicing is the process by which a precursor mRNA (pre-mRNA) can be transcribed from different RNA splicing methods; that is, different combinations of splice sites, to produce mutually exclusive mRNA splice isoforms, which in turn are translated to produce different protein products (Pan et al., 2008). This is the main function of RNA cyclization. Cyclization of circRNAs can be divided into intron and exon cyclization (Sanger et al., 1976), and the current mainstream cyclization mechanisms are categorized as follows: (1) exon skipping, (2) direct back-splicing of intron, (3) circRNA formation by RNA-binding proteins (RBPs; Chen, 2016; Zhang et al., 2018), and (4) circular intron RNA cyclization (Stoddard, 2014); the detailed mechanisms are shown in Figure 1. The diversity of circRNAs, and thus their diverse biological functions, is a direct result of these multiple formation mechanisms. For example, circRNAs can act as miRNA sponges (Hansen et al., 2013; Memczak et al., 2013; Zhao et al., 2020a), be translated into proteins (Yang et al., 2017), bind functional proteins (Li Z. et al., 2015), regulate RNA splicing (Conn et al., 2017), and regulate transcription (Chao et al., 1998; Memczak et al., 2013). Therefore, the identification of circRNAs contributes to our understanding of the formation and biological functions of circRNAs.

In 1976, Kolakofsky (1976) observed, for the first time, defective interfering RNAs in parainfluenza virus particles using electron microscopy. Sanger et al. (1976) discovered that plant-infecting viroids are a class of single-stranded, circular RNA molecules that have characteristics such as high thermal stability and a natural circular structure by self-complementary. In 1979, similar circular transcripts were found in HeLa cells and yeast mitochondria by electron microscopy (Hsu and Coca-Prados, 1979). In 1981, a ribosomal RNA (rRNA) gene was discovered in *Tetrahymena* that contained an intron sequence that formed a circular RNA after splicing. In 1988, the intron of 23S rRNA in archaea was found to be spliced at a specific site to form a stable circular RNA and to function as a transposon. In 1991, researchers identified several circular transcripts formed by different splicing patterns in the human oncogene DCC (Nigro et al., 1991), and these circular RNAs were then found in human *ETS1* gene, mouse *Sry* (sex-determining region Y) gene, rat cytochrome P450 *2C24* gene and human P450 *2C18* gene.

Despite their early discovery, research on circRNAs has been slow in recent decades. Although circRNAs were discovered decades ago, they could not be detected by molecular techniques that relied on poly(A) enrichment because they did not have free 3′ and 5′ ends. Instead, cyclizable exons were spliced by reverse splicing, which was different from regular linear splicing. Moreover, the mapping algorithm of early transcriptome analysis could not directly map the sequenced fragments to the genome, leading to the idea that circRNAs were byproducts of missplicing. With the development of high-throughput sequencing and bioinformatics technologies, it was first proposed in 2012 that circRNAs are circular transcripts generated by reverse splicing of mRNA precursors, which are found to exist in large quantities in different types of human cells. In 2013, it was found that circRNAs can act as a sponge for miRNAs (Hansen et al., 2013; Memczak et al., 2013), which regulate the growth and development of organisms. Since then, circRNAs have rapidly become a research hotspot. To identify circRNAs, in addition to high-throughput techniques (RNA-seq), common analytical and computational methods are used, such as CIRI (Gao et al., 2015), segemehl (Hoffmann et al., 2014), Mapsplice (Wang et al., 2010), and CircSeq (Guo et al., 2014). In recent years, researchers have developed machine learning methods to identify circRNAs based on the above methods (Yin et al., 2021). Feature selection is an important part of these machine learning models. Feature selection, aiming to select a subset of features by eliminating redundant and noise features, is an important preprocessing step in bioinformatics. Recently, Su et al. (2018) proposed a binomial distribution based method to perform feature selection in computational genomics. The effectiveness of their method has been proved by predicting lncRNA subcellular localizations (Su et al., 2018). Since both nucleotide and amino acid composition obey binomial distribution, this method is suggested to be used for genomic and proteomic analysis. We provide here an overview of the research progress of circRNAs, including the development of circRNA databases, identification of circRNAs, and the role of circRNAs in human diseases such as colon cancer, atherosclerosis, and gastric cancer.

## circRNA-Related Databases

In recent years, as circRNA research has progressed, an increasing number of circRNAs have been discovered in different species, and circRNA-related databases have been created. Some of the main circRNA databases published so far are listed below.

(1) circBase collects and merges public circRNA datasets and provides evidence of the genomic catalog of their expression, as well as scripts to identify circRNAs in sequencing data^1^ (Glazar et al., 2014).
(2) Circ2Trait is a comprehensive database that includes potential associations of circRNAs with diseases and traits by studying the interaction network of circRNAs with miRNAs and calculating their internal SNPs and Argonaute (Ago) interaction sites^2^ (Ghosal et al., 2013).
(3) deepBase contains about 150,000 circRNA genes from organisms, including human, mouse, *Drosophila*, and nematode. This database also constructs the most comprehensive expression map of circRNAs^3^ (Yang et al., 2010).
(4) CirNet mainly includes RNA-seq data of more than 400 samples from 26 tissues collected from the sequence read archive database. This database not only includes basic information on circRNAs but also provides expression profile data of circRNAs in different tissues and the competing endogenous (ce)RNA regulatory network of circRNAs–miRNA–gene^4^ (Liu et al., 2016).
(5) starBase v2.0 integrates published circRNA data and constructs interaction networks of miRNAs with circRNAs and circRNAs with RBPs. In addition, the database looks for potential miRNA–ncRNA, miRNA–mRNA, ncRNA–RNA, RBP–ncRNA, and RBP–mRNA interactions through high-throughput data. starBase also predicts the function of ncRNAs from miRNA-mediated (ceRNA) regulatory networks (miRNAs, lncRNAs, and pseudogenes) and protein-coding genes using the online tools miRFunction and ceRNAFunction^5^ (Li et al., 2014).

## Tools for Recognition of circRNAs

Because of the low expression level of circRNAs and limitations of previous computational methods, these RNA molecules were only found in small numbers in individual genes and therefore initially thought to be products of missplicing, byproducts of RNA splicing, incidental in animals, or precursors of linear RNAs. In recent years, with improved experimental and computational methods for circRNAs and the use of next-generation high-throughput sequencing technologies (Wang et al., 2009; Zeng et al., 2017, 2019), a large number of stable circRNAs have now been found in a variety of cells, and 85% of circRNAs can be mapped to known genes, of which 84% overlap with coding exons (Memczak et al., 2013). Because of the special structure of circRNAs—they lack a 5′ terminal cap and a 3′ terminal poly(A) tail and have a closed-loop structure with covalent bonds—and their maturation mechanism, early sequencing methods could not easily detect such molecules. Improvements in sequencing analysis techniques and computational methods have made detection more efficient (Malysiak-Mrozek et al., 2019; Mrozek, 2020). Therefore, studies on the identification of circRNAs are reviewed from two aspects: (1) identification based on sequencing data and (2) identification based on sequence features and machine learning methods.

### Identification of circRNAs Based on Sequencing

Many algorithms exist for circRNA identification, including CIRI (Gao et al., 2015), segemehl (Hoffmann et al., 2014), Mapsplice (Wang et al., 2010), CircSeq (Guo et al., 2014), and find_circ (Memczak et al., 2013). Using these algorithms, researchers have identified a large number of circRNAs in human, mouse, nematode, archaea, and other organisms (Yang et al., 2011; Jeck and Sharpless, 2014). We describe here several of these commonly used sequencing-based tools for identification of circRNAs.

CIRI (Stoddard, 2014) was developed by Gao et al. (2015) to comprehensively identify circRNAs, and it is based on the novel chiastic clipping signal algorithm. CIRI can accurately detect circRNAs from transcriptomic data without bias through multiple filtering strategies. This tool is mainly used to identify and annotate circRNAs from RNA-seq data. Unlike other methods for annotating circRNAs, CIRI eliminates false positives by using a new algorithm based on paired cross-clip signal detection in the BWA-MEM sequence alignment/map and combining it with systematic filtering.

CIRCexplorer, a tool for identifying circRNAs developed by Zhang et al. (2014), was the first to elucidate the regulatory mechanism of complementary sequences on production of exon-derived circRNAs. This tool revealed that regulation of variable cyclization was mediated by competitive pairing of complementary sequences, providing a new theoretical perspective on the complexity and diversity of gene expression at the transcriptional and posttranscriptional levels. Nearly 10,000 circRNAs were identified in human embryonic stem cell line H9 using a special nuclease to enrich circRNAs in combination with computational analysis software, demonstrating exon cyclization mediated by the complementary sequence of intron RNA. Competitive pairing of complementary sequences between different regions can selectively generate either linear RNAs or circRNAs.

CircSeq, a tool developed by Guo et al. (2014) to identify and characterize mammalian circRNAs, is a computational pipeline to identify and quantify the relative abundance of circRNAs from RNA-seq databases. Compared with other identification tools, CircSeq does not require available gene annotation to identify circRNAs. The application of the identification tool to non-polyA-selected RNA sequencing data in the ENCODE project proved its ability to classify and globally characterize more than 7000 human circRNAs.

The above sequencing methods all identify back-splicing sites from high-throughput sequencing data to detect circRNAs. In comparing some of the above identification tools, Hansen et al. (2016) and Sekar et al. (2019) found that only a small percentage of circRNAs could be predicted simultaneously by these tools, indicating significant differences and species variability. Therefore, the above tools developed around high-throughput sequencing technology have poor identification performance and low consistency. Moreover, these tools generally have high false-positive rates and low sensitivity (Hansen et al., 2016). To address these shortcomings, researchers have developed tools to identify circRNAs on the basis of sequence features and machine learning.

### Identification of circRNAs Based on Sequence Features and Machine Learning

**FIGURE 2:**
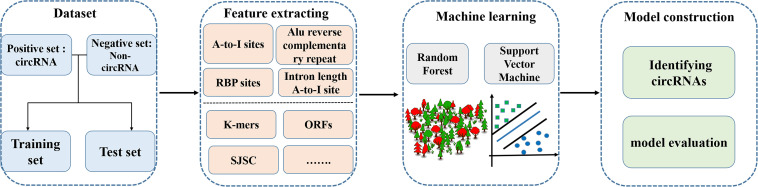
Methodology for predicting circRNAs based on machine learning methods.

Identifying circRNAs using sequence features that distinguish circRNAs from linear RNAs (especially mRNAs that encode proteins) is an urgent problem to be solved in bioinformatics. In recent years, the combination of sequence features and machine learning has been successfully used to solve biological problems such as the prediction of gene regulatory sites and splice sites (Wang et al., 2008; Xiong et al., 2015), and protein function (Cao et al., 2017; Gbenro et al., 2020; Hippe, 2020; Zhai et al., 2020), etc (Mrozek et al., 2007, 2009; Wei et al., 2017b,c, 2018; Jin et al., 2019; Stephenson et al., 2019; Su et al., 2019a,b; Liu B. et al., 2020; Liu Y. et al., 2020; Smith et al., 2020; Zhao et al., 2020b,c). Some tools have been developed to identify circRNAs using sequence features and machine learning methods. The basic framework of using machine learning methods to predict circRNAs is shown in Figure 2.

One study selected 100 RNA circularization-related sequence features, including length, adenosine-to-inosine (A-to-I) density, and Alu sequences of introns upstream and downstream of the splice site, and established a machine learning model to identify circRNAs in the human genome. The classification abilities of two machine learning methods, random forest (RF; Cheng et al., 2019b; Liu et al., 2019) and support vector machine (SVM; Jiang et al., 2013; Wei et al., 2014, 2017a, 2019; Zhao et al., 2015; Cheng, 2019; Hong et al., 2020; Li and Liu, 2020; Shao and Liu, 2020), were also compared. The results showed that the selected sequence features could effectively identify RNA circularization and that different sequence features contribute differently to the classification and prediction ability of the model. The RF method showed better classification than the SVM method.

In 2021, Yin et al. (2021) constructed a tool, named PCirc, to identify circRNAs using multiple sequence features and RF classification. This tool specifically targets the identification of circRNAs in plants, mainly from RNA sequence data. The tool encodes the sequence information of rice circRNAs by using three feature-encoding methods: k-mers, open reading frames, and splicing junction sequence coding (SJSC). The accuracy of the encoded information is greater than 80% when using the RF method for identification. The identification model can be used not only for the identification of rice circRNAs, but also for the recognition of circRNAs in plants such as *Arabidopsis thaliana*.

## circRNAs and Human Diseases

In terms of disease diagnosis, studies have found that the exosomes released by cancer cells contain abundant circRNAs, suggesting that circRNAs might be used as biological markers for clinical diagnosis. The key when using circRNAs for disease prediction is to identify the interaction site between the circRNA and miRNA or RBP, and then indirectly determine the association between the circRNA and disease by analyzing the relationship between the miRNA or RBP and disease (Jiang et al., 2010; Cheng et al., 2018; Liu, 2020; Zeng et al., 2020; Zuo et al., 2020).

In 2015, Li Y. et al. (2015) reported that exosomes are enriched with circRNAs, so it is possible that diseases such as colon cancer could be diagnosed by detecting circRNAs in serum. Aberrant expression of circRNAs in colorectal cancer and pancreatic ductal adenocarcinoma has been used as a diagnostic or predictive biomarker. By studying their expression profile, it was found that circRNAs may be associated with the molecular pathogenesis of cutaneous basal cell carcinoma (Sand et al., 2016).

The first validated circRNA, cANRIL, is closely related to a single nucleotide polymorphism (SNP) that is thought to alter the splicing of cANRIL, leading to expression of the *INK4A*/*ARF* loci, resulting in an increased incidence of atherosclerosis (Burd et al., 2010). Hypoxia is one of the key factors contributing to the development of atherosclerosis, and is therefore also regulated by circRNA (Boeckel et al., 2015).

Xu et al. (2015) showed that mice of a transgenic line overexpressing the *miR-7* gene in β-cells developed diabetes mellitus. The same study showed that overexpression of the circRNA ciRS-7 inhibited miR-7 function and thus improved insulin secretion. Potential target genes of *miR-7* have been identified by bioinformatics analysis and include *Myrip* (a gene regulating insulin secretory granules) and *Pax6* (a gene enhancing insulin transcription).

A study by Li P. et al. (2015) identified the circRNA hsa-circ002059 as being associated with gastric cancer. In that study, expression of this circRNA was downregulated in gastric tissues of patients compared with healthy controls. In addition, hsa-circ002059 was found at significantly lower levels in plasma of patients with gastric cancer than in healthy controls.

In bladder cancer, circRNAs have been identified using high-throughput microarray technology. Using this approach, Zhong et al. (2016) found two downregulated circRNAs (circFAM169A and circTRIM24) and 4 upregulated circRNAs (circTCF25, circZFR, circPTK2, and circBC048201) in bladder cancer tissue compared with adjacent non-tumor tissues. In addition, in the cancer tissues, circTCF25 could increase expression of the *CDK6* gene by modulating miR-103a-3p and miR-107. This is closely related to the development of cancer.

Qin et al. (2016) identified hsa-cir0001649 in hepatocellular carcinoma (HCC) and found that its expression was significantly decreased compared with that in adjacent normal liver tissue. In contrast, Shang et al. (2016) found that another circRNA, hsa-cir0005075, was significantly downregulated in HCC compared with adjacent normal tissue.

Exosomes are highly enriched with circRNAs. Exosomes are extracellular vesicles, 40 to 160 nm in diameter, that function as important intercellular signaling pathways (Li Y. et al., 2015; Kalluri and LeBleu, 2020). The exosome database exoRBase included 92 sequenced samples of serum exosomes, including samples from healthy volunteers and patients with coronary heart disease and colon cancer. The exosome samples contained 58,330 circRNAs and 18,333 mRNAs (Li et al., 2018). Zhang et al. (2019) demonstrated that circNRIP1, when secreted via exosome, can be taken up by gastric cancer cells and promote their proliferation, migration, and invasion. Therefore, exosomes can be regarded as *in vivo* carriers of circRNAs that can amplify their biological functions.

## Challenges and Prospects

Compared with long non-coding RNAs and miRNAs, research on circRNAs is still in its infancy and many questions remain to be answered, primarily in four areas:

(1) Transport and degradation: because circRNAs can resist RNase digestion and are stable in cells, the process of their degradation is unclear.
(2) Formation: it is unknown whether circRNAs are produced during or after transcription.
(3) Expression, translation, and function of circRNAs: circRNAs have stable structures and are highly conserved, underpinning their ability to play important roles in different organisms. Their unconfirmed roles, including acting as miRNA sponges, regulating gene expression, and targeting RBPs, require comprehensive and extensive elucidation.
(4) Research methodology: the experimental methodologies and bioinformatics used to identify circRNAs are challenging. For example, in experimental methods, general RNA-seq procedures such as reverse transcription may cause technical mis-ligation and generate a large number of artificial circRNAs. These pseudo circRNAs can account for 34–55% of the sequencing quantity, seriously affecting the accuracy of the data. As for methods that use machine learning and sequence features, only a few identification tools exist and their accuracy needs to be improved. These tools are not stable across different species. Therefore, in the future, stable identification models and deep learning methods are needed to establish identification tools for circRNAs and improve the robustness of the models.

Accurate identification will help determine additional biological functions of circRNAs. The unique features of circRNAs such as ceRNA may provide new ideas for drug discovery and development. The tissue specificity and stability of circRNAs make them potentially useful biomarkers. In the near future, it is likely that circRNAs will play important roles in the prevention, diagnosis, and treatment of various diseases.

## Author Contributions

ML and BG: conceptualization, writing—review and editing, and supervision. SJ, SH, and SW: investigation and writing—original draft preparation. All authors have read and agreed to the published version of the manuscript.

## Conflict of Interest

The authors declare that the research was conducted in the absence of any commercial or financial relationships that could be construed as a potential conflict of interest.

## Footnotes

1 http://www.circbase.org/

2 http://gyanxet-beta.com/circdb/

3 http://deepbase.sysu.edu

4 http://syslab5.nchu.edu.tw/CircNet

5 http://starbase.sysu.edu.cn/

## References

[B1] Boeckel J. N. Jae N. Heumueller A. W. Chen W. Boon R. A. Stellos K. (2015). Identification and. characterization of hypoxia-regulated endothelial circular RNA. *Circ. Res.* 117 884–890. 10.1161/CIRCRESAHA.115.306319 26377962

[B2] Burd C. E. Jeck W. R. Liu Y. Sanoff H. K. Wang Z. Sharpless N. E. (2010). Expression of linear and novel circular forms of an INK4/ARF-associated non-coding RNA correlates with atherosclerosis risk. *PLoS Genet.* 6:e1001233. 10.1371/journal.pgen.1001233 21151960 PMC2996334

[B3] Cao R. Freitas C. Chan L. Sun M. Jiang H. Chen Z. (2017). ProLanGO: protein function prediction using neural machine translation based on a recurrent neural network. *Molecules* 22:1732. 10.3390/molecules22101732 29039790 PMC6151571

[B4] Chao C. W. Chan D. C. Kuo A. Leder P. (1998). The mouse formin (Fmn) gene: abundant circular RNA transcripts and gene-targeted deletion analysis. *Mol. Med.* 4 614–628. 10.1007/bf03401761 9848078 PMC2230310

[B5] Chen L. L. (2016). The biogenesis and emerging roles of circular RNAs. *Nat. Rev. Mol. Cell Biol.* 17 205–211. 10.1038/nrm.2015.32 26908011

[B6] Cheng L. (2019). Computational and biological methods for gene therapy. *Curr. Gene Ther.* 19 210–210. 10.2174/156652321904191022113307 31762421

[B7] Cheng L. Hu Y. Sun J. Zhou M. Jiang Q. (2018). DincRNA: a comprehensive web-based bioinformatics toolkit for exploring disease associations and ncRNA function. *Bioinformatics* 34 1953–1956. 10.1093/bioinformatics/bty002 29365045

[B8] Cheng L. Wang P. Tian R. Wang S. Guo Q. Luo M. (2019). LncRNA2Target v2.0: a comprehensive database for target genes of lncRNAs in human and mouse. *Nucl. Acids Res.* 47 D140–D144. 30380072 10.1093/nar/gky1051 PMC6323902

[B9] Cheng L. Zhao H. Wang P. Zhou W. Luo M. Li T. (2019b). Computational methods for identifying similar diseases. *Mol. Ther. Nucl. Acids.* 18 590–604. 10.1016/j.omtn.2019.09.019 31678735 PMC6838934

[B10] Conn V. M. Hugouvieux V. Nayak A. Conos S. A. Capovilla G. Cildir G. (2017). A circRNA from SEPALLATA3 regulates splicing of its cognate mRNA through R-loop formation. *Nat. Plants* 3:17053. 10.1038/nplants.2017.53 28418376

[B11] Gao Y. Wang J. Zhao F. (2015). CIRI: an efficient and unbiased algorithm for de novo circular RNA identification. *Genome Biol.* 16:4. 10.1186/s13059-014-0571-3 PMC4316645 25583365

[B12] Gbenro S. Hippe K. Cao R. (2020). “HMMeta: Protein function prediction using hidden markov models,” in *Proceedings of the BCB ’20: 11th ACM International Conference on Bioinformatics, Computational Biology and Health Informatics* (New York, NY: Association for Computing Machinery).

[B13] Ghosal S. Das S. Sen R. Basak P. Chakrabarti J. (2013). Circ2Traits: a comprehensive database for circular RNA potentially associated with disease and traits. *Front. Genet.* 4:283. 10.3389/fgene.2013.00283 24339831 PMC3857533

[B14] Glazar P. Papavasileiou P. Rajewsky N. (2014). circBase: a database for circular RNAs. *RNA* 20 1666–1670. 10.1261/rna.043687.113 25234927 PMC4201819

[B15] Guo J. U. Agarwal V. Guo H. Bartel D. P. (2014). Expanded identification and characterization of mammalian circular RNAs. *Genome Biol.* 15:409. 10.1186/s13059-014-0409-z PMC4165365 25070500

[B16] Hansen T. B. Jensen T. I. Clausen B. H. Bramsen J. B. Finsen B. Damgaard C. K. (2013). Natural RNA circles function as efficient microRNA sponges. *Nature* 495 384–388. 10.1038/nature11993 23446346

[B17] Hansen T. B. Veno M. T. Damgaard C. K. Kjems J. (2016). Comparison of circular RNA prediction tools. *Nucl. Acids Res.* 44:e58. 10.1093/nar/gkv1458 26657634 PMC4824091

[B18] Hippe K. (2020). “Sola gbenro; renzhi cao in *prolango2: protein function prediction with ensemble of encoder-decoder networks*,” in *Proceedings of the BCB ’20: 11th ACM International Conference on Bioinformatics, Computational Biology and Health Informatics* (New York, NY: Association for Computing Machinery).

[B19] Hoffmann S. Otto C. Doose G. Tanzer A. Langenberger D. Christ S. (2014). A multi-split mapping algorithm for circular RNA, splicing, trans-splicing and fusion detection. *Genome Biol.* 15:R34. 10.1186/gb-2014-15-2-r34 PMC4056463 24512684

[B20] Hong Z. Zeng X. Wei L. Liu X. (2020). Identifying enhancer-promoter interactions with neural network based on pre-trained DNA vectors and attention mechanism. *Bioinformatics* 36 1037–1043. 31588505 10.1093/bioinformatics/btz694

[B21] Hsu M. T. Coca-Prados M. (1979). Electron microscopic evidence for the circular form of RNA in the cytoplasm of eukaryotic cells. *Nature* 280 339–340. 10.1038/280339a0 460409

[B22] Jeck W. R. Sharpless N. E. (2014). Detecting and characterizing circular RNAs. *Nat. Biotechnol.* 32 453–461. 10.1038/nbt.2890 24811520 PMC4121655

[B23] Jiang Q. Hao Y. Wang G. Juan L. Zhang T. Teng M. (2010). Prioritization of disease microRNAs through a human phenome-microRNAome network. *BMC Syst. Biol.* 4(Suppl. 1):S2. 10.1186/1752-0509-4-S1-S2 20522252 PMC2880408

[B24] Jiang Q. Ma R. Wang J. Wu X. Jin S. Peng J. (2015). LncRNA2Function: a comprehensive resource for functional investigation of human lncRNAs based on RNA-seq data. *BMC Genomics.* 16(Suppl. 3):S2. 10.1186/1471-2164-16-S3-S2 25707511 PMC4331805

[B25] Jiang Q. Wang G. Jin S. Li Y. Wang Y. (2013). Predicting human microRNA-disease associations based on support vector machine. *Int. J. Data Min. Bioinform.* 8 282–293. 10.1504/ijdmb.2013.056078 24417022

[B26] Jiang Q. Wang J. Wang Y. Ma R. Wu X. Li Y. (2014). TF2LncRNA: identifying common transcription factors for a list of lncRNA genes from ChIP-Seq data. *Biomed Res. Int.* 2014:317642. 10.1155/2014/317642 PMC3960524 24729968

[B27] Jiang Q. Wang Y. Hao Y. Juan L. Teng M. Zhang X. (2009). miR2Disease: a manually curated database for microRNA deregulation in human disease. *Nucl. Acids Res.* 37 D98–D104. 18927107 10.1093/nar/gkn714 PMC2686559

[B28] Jin Q. Meng Z. Tuan D. P. Chen Q. Wei L. Su R. (2019). DUNet: a deformable. *Knowl. Based Syst.* 178 149–162. 10.1016/j.knosys.2019.04.025

[B29] Kalluri R. LeBleu V. S. (2020). The biology, function, and biomedical applications of exosomes. *Science* 367:eaau6977. 10.1126/science.aau6977 32029601 PMC7717626

[B30] Kolakofsky D. (1976). Isolation and characterization of Sendai virus DI-RNAs. *Cell* 8 547–555. 10.1016/0092-8674(76)90223-3 182384

[B31] Li C. C. Liu B. (2020). MotifCNN-fold: protein fold recognition based on fold-specific features extracted by motif-based convolutional neural networks. *Brief. Bioinform.* 21 2133–2141. 10.1093/bib/bbz133 31774907

[B32] Li J. H. Liu S. Zhou H. Qu L. H. Yang J. H. (2014). starBase v2.0: decoding miRNA-ceRNA, miRNA-ncRNA and protein-RNA interaction networks from large-scale CLIP-Seq data. *Nucl. Acids Res.* 42 D92–D97. 24297251 10.1093/nar/gkt1248 PMC3964941

[B33] Li P. Chen S. Chen H. Mo X. Li T. Shao Y. (2015). Using circular RNA as a novel type of biomarker in the screening of gastric cancer. *Clin. Chim. Acta* 444 132–136. 10.1016/j.cca.2015.02.018 25689795

[B34] Li S. Li Y. Chen B. Zhao J. Yu S. Tang Y. (2018). exoRBase: a database of circRNA, lncRNA and mRNA in human blood exosomes. *Nucl. Acids Res.* 46 D106–D112. 30053265 10.1093/nar/gkx891 PMC5753357

[B35] Li Y. Zheng Q. Bao C. Li S. Guo W. Zhao J. (2015). Circular RNA is enriched and stable in exosomes: a promising biomarker for cancer diagnosis. *Cell Res.* 25 981–984. 10.1038/cr.2015.82 26138677 PMC4528056

[B36] Li Z. Huang C. Bao C. Chen L. Lin M. Wang X. (2015). Exon-intron circular RNAs regulate transcription in the nucleus. *Nat. Struct. Mol. Biol.* 22 256–264. 10.1038/nsmb.2959 25664725

[B37] Liang C. Changlu Q. He Z. Tongze F. Xue Z. (2019). gutMDisorder: a comprehensive database for dysbiosis of the gut microbiota in disorders and interventions. *Nucl. Acids Res.* 48:7603. 10.1093/nar/gkaa511 PMC7367202 32515792

[B38] Liu B. Gao X. Zhang H. (2019). BioSeq-analysis2.0: an updated platform for analyzing DNA, RNA, and protein sequences at sequence level and residue level based on machine learning approaches. *Nucl. Acids Res.* 47:e127. 10.1093/nar/gkz740 31504851 PMC6847461

[B39] Liu B. Zhu Y. Yan K. (2020). Fold-LTR-TCP: protein fold recognition based on triadic closure principle. *Brief. Bioinform.* 21 2185–2193. 10.1093/bib/bbz139 31813954

[B40] Liu Y. C. Li J. R. Sun C. H. Andrews E. Chao R. F. Lin F. M. (2016). CircNet: a database of circular RNAs derived from transcriptome sequencing data. *Nucl. Acids Res.* 44 D209–D215. 26450965 10.1093/nar/gkv940 PMC4702939

[B41] Liu Y. Huang Y. Wang G. Wang Y. (2020). A deep learning approach for filtering structural variants in short read sequencing data. *Brief Bioinform.* 10.1093/bib/bbaa370 33378767

[B42] Liu Z. P. (2020). Predicting lncRNA-protein interactions by machine learning methods: a review. *Curr. Bioinform.* 15 831–840. 10.2174/1574893615666200224095925

[B43] Malysiak-Mrozek B. Baron T. Mrozek D. (2019). Spark-IDPP: high-throughput and scalable prediction of intrinsically disordered protein regions with Spark clusters on the cloud. *Cluster Comput. J. Net. Softw. Tools Appl.* 22 487–508. 10.1007/s10586-018-2857-9

[B44] Memczak S. Jens M. Elefsinioti A. Torti F. Krueger J. Rybak A. (2013). F le noble., N rajewsky, circular RNAs are a large class of animal RNAs with regulatory potency. *Nature* 495 333–338. 10.1038/nature11928 23446348

[B45] Mrozek D. (2020). A review of cloud computing technologies for comprehensive microRNA analyses. *Comput. Biol. Chem.* 88:107365. 10.1016/j.compbiolchem.2020.107365 32906056

[B46] Mrozek D. Malysiak B. Kozielski S. (2007). “An optimal alignment of proteins energy characteristics with crisp and fuzzy similarity awards,” in *Proceedings of the****2007 Ieee International Conference on Fuzzy Systems***, Vol. 1-4 (London: IEEE), 1513–1518.

[B47] Mrozek D. Malysiak-Mrozek B. Kozielski S. (2009). *Alignment of Protein Structure Energy Patterns Represented as Sequences of Fuzzy Numbers.* Cincinnati, OH: IEEE, 35–40.

[B48] Nigro J. M. Cho K. R. Fearon E. R. Kern S. E. Ruppert J. M. Oliner J. D. (1991). Scrambled exons. *Cell.* 64 607–613. 10.1016/0092-8674(91)90244-s 1991322

[B49] Pan Q. Shai O. Lee L. J. Frey J. Blencowe B. J. (2008). Deep surveying of alternative splicing complexity in the human transcriptome by high-throughput sequencing. *Nat. Genet.* 40 1413–1415. 10.1038/ng.259 18978789

[B50] Pradeep C. Nandan D. Das A. A. Velayutham D. (2020). Comparative transcriptome profiling of disruptive technology, single-molecule direct RNA sequencing. *Curr. Bioinf.* 15 165–172. 10.2174/1574893614666191017154427

[B51] Qin M. Liu G. Huo X. Tao X. Sun X. Ge Z. (2016). Hsa_circ_0001649: a circular RNA and potential novel biomarker for hepatocellular carcinoma. *Cancer Biomark.* 16 161–169. 26600397 10.3233/CBM-150552 PMC13016540

[B52] Salgia S. R. Singh S. K. Gurha P. Gupta R. (2003). Two reactions of *Haloferax voicanii* RNA splicing enzymes: joining of exons and circularization of introns. *RNA* 9 319–330. 10.1261/rna.2118203 12592006 PMC1370399

[B53] Sand M. Bechara F. G. Sand D. Gambichler T. Hahn S. A. Bromba M. (2016). Circular RNA expression in basal cell carcinoma. *Epigenomics* 8 619–632. 10.2217/epi-2015-0019 27097056

[B54] Sanger H. L. Klotz G. Riesner D. Gross H. J. Kleinschmidt A. K. (1976). Viroids are single-stranded covalently closed circular RNA molecules existing as highly base-paired rod-like structures. *PNAS* 73 3852–3856. 10.1073/pnas.73.11.3852 1069269 PMC431239

[B55] Sekar S. Geiger P. Cuyugan L. Boyle A. Serrano G. Beach T. G. (2019). Identification of circular RNAs using RNA sequencing. *J. Vis. Exp.* 14:e59981. 10.3791/59981 31789321

[B56] Shang X. Li G. Liu H. Li T. Liu J. Zhao Q., et al. (2016). Comprehensive circular RNA profiling reveals that hsa_circ_0005075, a new circular RNA biomarker, is involved in hepatocellular crcinoma development. *Medicine* 95:e3811. 10.1097/MD.0000000000003811 PMC4900729 27258521

[B57] Shao J. Liu B. (2020). ProtFold-DFG: protein fold recognition by combining directed fusion graph and pagerank algorithm. *Brief. Bioinform.* 10.1093/bib/bbaa192 32892224

[B58] Smith J. Conover M. Stephenson N. Eickholt J. Si D. Sun M. (2020). TopQA: a topological representation for single-model protein quality assessment with machine learning. *J. Int. J. Comput. Biol. Drug Des.* 13:144. 10.1504/ijcbdd.2020.10026784

[B59] Stephenson N. Shane E. Chase J. Rowland J. Ries D. Justice N. (2019). Survey of machine learning techniques in drug discovery. *Curr. Drug Metab.* 20 185–193. 10.2174/1389200219666180820112457 30124147

[B60] Stoddard B. L. (2014). Homing endonucleases from mobile group I introns: discovery to genome engineering. *Mobile DNA* 5:7. 10.1186/1759-8753-5-7 24589358 PMC3943268

[B61] Su R. Liu X. Wei L. Zou Q. (2019a). Deep-Resp-Forest: a deep forest model to predict anti-cancer drug response. *Methods (San Diego, Calif.)* 166 91–102. 10.1016/j.ymeth.2019.02.009 30772464

[B62] Su R. Wu H. Xu B. Liu X. Wei L. (2019b). Developing a multi-dose computational model for drug-induced hepatotoxicity prediction based on toxicogenomics data. *IEEE-ACM Trans. Comput. Biol. Bioinform.* 16 1231–1239. 10.1109/tcbb.2018.2858756 30040651

[B63] Su Z. D. Huang Y. Zhang Z. Y. Zhao Y. W. Wang D. Chen W. (2018). iLoc-lncRNA: predict the subcellular location of lncRNAs by incorporating octamer composition into general PseKNC. *Bioinformatics* 34 4196–4204. 29931187 10.1093/bioinformatics/bty508

[B64] Wang G. Wang Y. Feng W. Wang X. Yang J. Y. Zhao Y. (2008). Transcription factor and microRNA regulation in androgen-dependent and -independent prostate cancer cells. *BMC Genom.* 9 (Suppl. 2):S22. 10.1186/1471-2164-9-S2-S22 18831788 PMC2559887

[B65] Wang K. Singh D. Zeng Z. Coleman S. J. Huang Y. Savich G. L. (2010). MapSplice: accurate mapping of RNA-seq reads for splice junction discovery. *Nucl. Acids Res.* 38:e178. 10.1093/nar/gkq622 20802226 PMC2952873

[B66] Wang P. L. Bao Y. Yee M. C. Barrett S. P. Hogan G. J. Olsen M. N. (2014). Circular RNA is expressed across the eukaryotic tree of life. *PLoS One* 9:e90859. 10.1371/journal.pone.0090859 24609083 PMC3946582

[B67] Wang Z. Gerstein M. Snyder M. (2009). RNA-Seq: a revolutionary. *Nat. Rev. Genet.* 10 57–63. 19015660 10.1038/nrg2484 PMC2949280

[B68] Wei H. Liu B. (2020). iCircDA-MF: identification of circRNA-disease associations based on matrix factorization. *Brief. Bioinform.* 21 1356–1367. 10.1093/bib/bbz057 31197324

[B69] Wei L. Ding Y. Su R. Tang J. Zou Q. (2018). Prediction of human protein subcellular localization using deep learning. *J. Parallel Distrib. Comput.* 117 212–217.

[B70] Wei L. Liao M. Gao Y. Ji R. He Z. Zou Q. (2014). Improved and promising identification of human MicroRNAs by incorporating a high-quality negative set. *IEEE/ACM Trans. Comput. Biol. Bioinform.* 11 192–201. 10.1109/tcbb.2013.146 26355518

[B71] Wei L. Tang J. Zou Q. (2017a). Local-DPP: an improved DNA-binding protein prediction method by exploring local evolutionary information. *Inf. Sci.* 384 135–144. 10.1016/j.ins.2016.06.026

[B72] Wei L. Wan S. Guo J. Wong K. K. L. (2017c). A novel hierarchical selective ensemble classifier with bioinformatics application. *Artif. Intell. Med.* 83 82–90. 10.1016/j.artmed.2017.02.005 28245947

[B73] Wei L. Xing P. Shi G. Ji Z. Zou Q. (2019). Fast prediction of protein methylation sites using a sequence-based feature selection technique. *IEEE-ACM Trans. Comput. Biol. Bioinform.* 16 1264–1273. 10.1109/tcbb.2017.2670558 28222000

[B74] Wei L. Xing P. Zeng J. Chen J. X. Su R. Guo F. (2017b). Improved prediction of protein-protein interactions using novel negative samples, features, and an ensemble classifier. *Artif. Intell. Med.* 83 67–74. 10.1016/j.artmed.2017.03.001 28320624

[B75] Xiong H. Y. Alipanahi B. Lee L. J. Bretschneider H. Merico D. Yuen R. K. C. (2015). RNA splicing. the human splicing code reveals new insights into the genetic determinants of disease. *Science* 347 1254806. 10.1126/science.1254806 PMC4362528 25525159

[B76] Xu H. Guo S. Li W. Yu P. (2015). The circular RNA Cdr1as, via miR-7 and its targets, regulates insulin transcription and secretion in islet cells. *Sci. Rep.* 5:12453. 10.1038/srep12453 PMC4515639 26211738

[B77] Yang J. H. Shao P. Zhou H. Chen Y. Q. Qu L. H. (2010). deepBase: a database for deeply annotating and mining deep sequencing data. *Nucl. Acids Res.* 38 D123–D130. 19966272 10.1093/nar/gkp943 PMC2808990

[B78] Yang L. Duff M. O. Graveley B. R. Carmichael G. G. Chen L. L. (2011). Genomewide characterization of non-polyadenylated RNAs. *Genome Biol.* 12:R16. 10.1186/gb-2011-12-2-r16 PMC3188798 21324177

[B79] Yang Q. Wu J. Zhao J. Xu T. Han P. Song X. (2020). The expression profiles of lncrnas and their regulatory network during smek1/2 knockout mouse neural stem cells differentiation. *Curr. Bioinform.* 15 77–88. 10.2174/1574893614666190308160507

[B80] Yang Y. Fan X. Mao M. Song X. Wu P. Zhang Y. (2017). Extensive translation of circular RNAs driven by N-6-methyladenosine. *Cell Res.* 27 626–641. 10.1038/cr.2017.31 28281539 PMC5520850

[B81] Yin S. Tian X. Zhang J. Sun P. Li G. (2021). PCirc: random forest-based plant circRNA identification software. *BMC Bioinf.* 22:10. 10.1186/s12859-020-03944-1 33407069 PMC7789375

[B82] Zeng X. X. Lin W. Guo M. Z. Zou Q. (2019). Details in the evaluation of circular RNA detection tools: reply to Chen and Chuang. *PLoS Comput. Biol.* 15:5. 10.1371/journal.pcbi.1006916 31022173 PMC6527241

[B83] Zeng X. Lin W. Guo M. Zou Q. (2017). A comprehensive overview and evaluation of circular RNA detection tools. *PLoS Comput. Biol.* 13:e1005420. 10.1371/journal.pcbi.1005420 28594838 PMC5466358

[B84] Zeng X. Zhong Y. Lin W. Zou Q. (2020). Predicting disease-associated circular rnas using deep forests combined with positive-unlabeled learning methods. *Brief. Bioinform.* 21 1425–1436. 10.1093/bib/bbz080 31612203

[B85] Zhai Y. Chen Y. Teng Z. Zhao Y. (2020). Identifying antioxidant proteins by using amino acid composition and protein-protein interactions. *Front. Cell Dev. Biol.* 8:591487. 10.3389/fcell.2020.591487 33195258 PMC7658297

[B86] Zhang X. Wang S. Wang H. Cao J. Huang X. Chen Z. (2019). Circular RNA circNRIP1 acts as a microRNA-149-5p sponge to promote gastric cancer progression via the AKT1/mTOR pathway. *Mol. Cancer* 18:20. 10.1186/s12943-018-0935-5 PMC6360801 30717751

[B87] Zhang X.-Q. Wang H.-B. Zhang Y. Lu X. Chen L.-L. Yang L. (2014). Complementary sequence-mediated exon circularization. *Cell* 159 134–147. 10.1016/j.cell.2014.09.001 25242744

[B88] Zhang Z. Yang T. Xiao J. (2018). Circular RNAs: promising biomarkers for human diseases. *Ebiomedicine* 34 267–274. 10.1016/j.ebiom.2018.07.036 30078734 PMC6116471

[B89] Zhao T. Hu Y. Cheng L. (2020a). Deep-DRM: a computational method for identifying disease-related metabolites based on graph deep learning approaches. *Brief. Bioinform.* 10.1093/bib/bbaa212 33048110

[B90] Zhao T. Hu Y. Peng J. Cheng L. (2020b). DeepLGP: a novel deep learning method for prioritizing lncRNA target genes. *Bioinformatics* 36 4466–4472. 10.1093/bioinformatics/btaa428 32467970

[B91] Zhao X. Jiao Q. Li H. Wu Y. Wang H. Huang S. (2020c). ECFS-DEA: an. ensemble classifier-based feature selection for differential expression analysis on expression profiles. *BMC Bioinformatics* 21:43. 10.1186/s12859-020-3388-y PMC7003361 32024464

[B92] Zhao Y. Wang F. Juan L. (2015). MicroRNA promoter identification in arabidopsis using multiple histone markers. *Biomed Res. Int.* 2015:861402. 10.1155/2015/861402 PMC4573627 26425556

[B93] Zhong Z. Lv M. Chen J. (2016). Screening differential circular RNA expression profiles reveals the regulatory role of circTCF25-miR-103a-3p/miR-107-CDK6 pathway in bladder carcinoma. *Sci. Rep.* 6:30919. 10.1038/srep30919 PMC4971518 27484176

[B94] Zuo Y. Zou Q. Li J. Jiang M. Liu X. (2020). 2lpiRNApred: a two-layered integrated algorithm for identifying piRNAs and their functions based on LFE-GM feature selection. *RNA biology* 17 892–902. 10.1080/15476286.2020.1734382 32138598 PMC7549647

